# Increased Biodiversity in the Environment Improves the Humoral Response of Rats

**DOI:** 10.1371/journal.pone.0120255

**Published:** 2015-04-08

**Authors:** Cinthia Pi, Emma H. Allott, Daniel Ren, Susan Poulton, S. Y. Ryan Lee, Sarah Perkins, Mary Lou Everett, Zoie E. Holzknecht, Shu S. Lin, William Parker

**Affiliations:** 1 Department of Surgery, Duke University Medical Center, Durham, NC, United States of America; 2 Lineberger Comprehensive Cancer Center, University of North Carolina at Chapel Hill, Chapel Hill, NC, United States of America; 3 Cardiff School of Biosciences, Biomedical Sciences Building, Museum Avenue, Cardiff, United Kingdom; University of Maryland School of Medicine, UNITED STATES

## Abstract

Previous studies have compared the immune systems of wild and of laboratory rodents in an effort to determine how laboratory rodents differ from their naturally occurring relatives. This comparison serves as an indicator of what sorts of changes might exist between modern humans living in Western culture compared to our hunter-gatherer ancestors. However, immunological experiments on wild-caught animals are difficult and potentially confounded by increased levels of stress in the captive animals. In this study, the humoral immune responses of laboratory rats in a traditional laboratory environment and in an environment with enriched biodiversity were examined following immunization with a panel of antigens. Biodiversity enrichment included colonization of the laboratory animals with helminths and co-housing the laboratory animals with wild-caught rats. Increased biodiversity did not apparently affect the IgE response to peanut antigens following immunization with those antigens. However, animals housed in the enriched biodiversity setting demonstrated an increased mean humoral response to T-independent and T-dependent antigens and increased levels of “natural” antibodies directed at a xenogeneic protein and at an autologous tissue extract that were not used as immunogens.

## Introduction

We and others have previously compared the immune systems of wild rodents with that of laboratory rodents [1–4]. The studies provide one way of accessing the effect of laboratory environments on immune function in rodents. Of potential medical importance, this comparison can serve as a model for comparing the immune systems of humans in a hunter-gatherer environment with humans in a modern, Westernized environment. The studies have provided a trove of information, uncovering a number of mechanisms by which the immune systems of wild rats are much differently regulated than that of laboratory rats. For example, low levels of “natural” antibodies were found in laboratory rats compared to wild rats [5]. This finding has implications for the progression of cancer in biome depleted environments, since natural antibodies are important for tumor surveillance [6, 7]. However, those studies have some limitations inherent in immunological studies utilizing wild caught rodents. Not only are the genetics of the wild-caught animals poorly defined, but experiments on the animals involving multiple procedures and captivity are impractical due to the extreme stress induced by captivity and the potential effects of that stress on immune function.

We sought to further define the effects of the laboratory environment on immune function, but rather than using wild-caught rats for comparison with laboratory animals, we utilized laboratory animals which had been exposed to a “wild-like” environment. This wild-like environment, with greatly increased biodiversity (biome enriched) compared to the laboratory setting (biome depleted), included inoculation of the animals with helminths, co-housing with wild-caught rats, and the introduction of bedding from unregulated rodent facilities. This approach has considerable advantages over using wild-caught rodents in terms of isolating the variable of biodiversity. In particular, genetic differences between cohorts of animals are eliminated, and variation in factors such as diet, exercise and stress are minimized.

The model we utilized is less than ideal in terms of defining how exactly specific symbionts alter immune function. Indeed, it would be difficult if not impossible to define all of the changes, some of which might be transient, in the biodiversity of the wild-like environment. However, the wild-like model is very useful for examining the general role of biodiversity in immune function and, as stated above, has several advantages over our previous experiments using wild-caught animals.

Because the wild-like environment utilizes domesticated, laboratory rodents rather than wild-caught rodents, experiments involving multiple procedures and long-term captivity are feasible. With this in mind, we evaluated the humoral response of laboratory rats in a traditional laboratory setting (biome depleted) and in the wild-like environment (biome enriched). The response to a series of immunizations, including known allergens, T-dependent antigens and T-independent antigens in the two groups of animals was compared.

## Methods

### Standard laboratory conditions (biome depleted) and “biome enriched” conditions

All experiments were approved by the Duke University Institutional Animal Care and Use Committee. Male (n = 4) and female (n = 8) Sprague Dawley rats from Harlan Sprague Dawley (Indianapolis, IN, USA) were housed in a standard (hygienic) laboratory setting, except that cages were modified to accommodate the experiment. Specifically, the plastic sides of the traditional cages were replaced with wire so that free exchange between the animals and their environment could occur. Although this modification was necessary only in the biome enriched environment, the same cage system was used in the traditional laboratory setting (biome depleted) to avoid any potential effects of housing from confounding the comparison between the two groups. Cages consisted of a 40.6 cm high box with floor dimensions of 61 cm by 35.5 cm. The sides and top were constructed of 1.27 cm by 2.54 cm galvanized steel mesh, and a drop-in 7.62 cm deep plastic pan was used as the flooring. After acclimatization for 62 days, the animals were bred, yielding 31 female F_1_ rats (male F_1_ animals were not used in the experiment). Each F_1_ female rat was weighed at 4 days of age and again at 23 days of age. The animals were weaned at 23 days of age. Twenty of the 31 female F_1_ rats were selected at random and immunized according to the protocol described below.

In addition to the 12 laboratory rats housed under standard laboratory conditions (described above, with the exception of the cages), 12 additional Sprague Dawley rats (Harlan Sprague Dawley, 4 male and 8 female) were introduced into a facility for biome enrichment that was located in a building not used for the housing of any other laboratory rodents. Housing conditions, including temperature, lighting, cage construction, food and water were identical to those in standard laboratory housing. In this setting, the F_0_ rats were exposed to three sources of potential biome enrichment:

1. Nine days before arrival of the F_0_ rats, four female wild rats (*Rattus Norwegicus*) were caught in live traps in Durham, North Carolina and housed in the facility. Upon arrival, 62 days before breeding, the F_0_ laboratory rats were placed in cages next to the wild rats, and used bedding from the wild rat cages was introduced weekly into the cages housing the F_0_ laboratory rats.
2. After arrival at the animal facility, the F_0_ rats were exposed to bedding, refreshed weekly, from rats housed under non-standard (no barrier or regular screening) conditions from a commercial pet supplier.
3. 56 days before breeding, each F_0_ female was fed 3 *Hymenolepis diminuta* cysticercoids (the larval state of the rat tapeworm, a helminth) in a drop of 0.6% saline. The cysticercoids were harvested from meal bugs containing the organisms (Carolina Biological Supply, Burlington, NC, USA) using a Hund Wetzlar Wilovert dissecting microscope. This approach resulted in confirmed colonization (confirmed using a modified version of the McMaster technique) with *Hymenolepis diminuta* of 2 out of the 3 female F_0_ rats used in this study. (Of the original 8 F_0_ females, two animals were sacrificed prior to breeding and three others did not get pregnant during breeding; see below.) Confirmation of the presence or absence of helminth colonization was performed 3 weeks after exposure to *Hymenolepis diminuta* cysticercoids, and was repeated three times. Although *Hymenolepis diminuta* readily colonizes laboratory rats and will survive for the lifespan of those rats [8], it remains unknown why the organisms failed to colonize one of the rats under biome-enriched conditions, and it is unknown how long the helminths survived under those conditions.

At the biome enrichment facility, one F_0_ male and two F_0_ females were sacrificed due to apparent respiratory difficulty and weight loss. Breeding of the 6 remaining F_0_ female rats resulted in 3 pregnant female F_0_ rats and 15 female F_1_ rats. We subsequently (in a study unrelated to the present work) determined that removal of the commercial bedding improved the rate of effective breeding, suggesting that factors (e.g., scent from older males) present in the commercial bedding may have interfered with the willingness of the animals to breed. The wild rats remained in the facility for the duration of the experiment, although bedding from the wild rats and bedding from other non-clean facilities were not transferred to the cages of the F_1_ laboratory rats after weaning at 23 days of age. Further, F_1_ laboratory rats were not intentionally colonized with helminths; *Hymenolepis diminuta* requires an intermediate host (an insect containing the cysticercoid that is ingested by the primary host) for completion of its life cycle, so it is unlikely that the organism is an important component of the biome in mammals prior to weaning, before the animals forage. However, the F1 animals may have acquired the helminths naturally after weaning, and the extent of biome enrichment with *Hymenolepis diminuta* or with a wide range of other potential symbionts was not quantified (see Discussion). All 15 of the female F_1_ rats in the biome enrichment facility were immunized according to the protocol described below.

### Labeling of keyhole limpet hemocyanin (KLH) and bovine serum albumin (BSA) with fluorescein isothiocyanate (FITC)

Fluorescein was covalently coupled to KLH and to BSA to provide a T-dependent antigen for immunization and an antigen for use in ELISA assays, respectively. To label KLH (Sigma-Aldrich, St. Louis, MO, USA) with FITC (Sigma-Aldrich, St. Louis, MO, USA), 1.5mL of 10mg/mL KLH was first dialyzed against 2L of 100mM NaCO_3_ buffer at pH 9.3 in dialysis tubing (MWCO: 3,500; VWR Scientific, Rancho Dominguez, CA, USA) at 4°C for 24 hrs with one change in dialysis solution during this process. FITC and KLH were then combined at a 20μg:1mg ratio, and incubated for 1.5 h at room temperature. Next, the FITC-KLH conjugate was dialyzed in 500mL saline solution overnight at 4°C. The buffer was changed, and the sample was dialyzed for 2 hrs at room temperature. The absorbance of purified FITC-KLH was read at 280nm and 495nm, and the labeling ratio (FITC:Protein) was determined based on the ratio of those absorbances. Two batches, with labeling ratios 2.4 and 5.3, were made and pooled. BSA (Roche, Indianapolis, IN, USA) was labeled using the same protocol, with a molecular labeling ratio of 1:4. Both labeled proteins, FITC-KLH and FITC-BSA, were stored at -80°C until use. Labeled proteins were stored so that each aliquot was thawed only once prior to use.

### Peanut antigen extraction

A peanut extract was prepared in order to provide an immunogen that would elicit an IgE response upon injection. For this purpose, a peanut extract was prepared according to the method of deJonge et al. [9] with minor modifications: Raw peanuts were lightly blanched at 100°C for 3 min, pulverized using a BioPulverizer (Biospec Products, Inc. Bartlesville, OK, USA) and mixed with phosphate buffered saline (Roche, Indianapolis, IN, USA) at a 1kg:4L ratio. A homogenizer (OMNI GLH) was used to further homogenize the peanut-PBS mixture. The mixture was stirred overnight at 4°C, then centrifuged for 30 min at 100 x g at room temperature. The aqueous fraction was centrifuged for 30 more min at 100 x g at room temperature to remove the residual lipids. The remaining aqueous fraction, or “peanut extract”, was collected and stored at -80°C until use. The protein concentration was determined with the Pierce BCA Protein Assay Kit (Thermo Scientific, Rockford, IL, USA) according to manufacturer’s instructions. The extract was stored so that each aliquot was thawed only once prior to use.

### Immunization with peanut extract, FITC-KLH, and DNP-Ficoll

Biome depleted and biome enriched F_1_ rats (n = 20 and 15, respectively) were immunized with peanut extract, FITC-KLH, and DNP-Ficoll (2,4 Dinitrophenyl conjugated to AminoEthylCarboxyMethyl-Ficoll, a high molecular weight polysaccharide; Biosearch Technologies, Inc., Novato, CA, USA) according to the following protocol. An immunization “cocktail” was prepared by thawing and mixing peanut extract, FITC-KLH, and DNP-Ficoll at a concentrations of 2mg/mL:1mg/mL:2mg/mL, respectively. The cocktail was aliquoted and stored at -80°C until use. On Day 0, a portion of the cocktail was thawed, combined with Imject Alum (Pierce, Rockford, IL, USA) in a 1:1 (v/v) ratio, and mixed for 30 min on an Everlast Rocker at medium setting at room temperature. Rats were weighed (Day 0, age 43 days old), and peanut extract, FITC-KLH, and DNP-Ficoll emulsified with alum were injected intraperitoneally at a dose of 2mg/kg, 1mg/kg, and 2mg/kg, respectively. On days 2, 4, 7, and 9, the peanut extract (alone, without other antigens) was injected intraperitoneally at 2 mg/kg (using the Day 0 weight). On day 14, rats were injected with the immunization cocktail in a manner similar to that on Day 0, except that the alum conjugate was not included. On day 28 of the immunization protocol, when the F_1_ rats were 71 days old, the animals were euthanized with CO_2_ and blood was collected from the posterior vena cava. Blood samples were centrifuged in BD Vacutainer Plus blood collection tubes (BD Biosciences, Franklin Lakes, NJ, USA) at 2390 x g at 22°C for 10 min, and the sera was stored at -80°C until use.

### Measurement of Ig concentrations by ELISA

ELISA assays were run under conditions in which a linear relationship existed between the serum concentration and the absorbance readout. Initial dilution series were run for each antibody to determine this “linear range”. Assays for total IgE and anti-peanut IgE employed a dilution of 1:800. All other ELISA assays employed a dilution of 1:50. All assays were run in duplicate except for the sera standard on each plate, which was run in quadruplicate.

Relative concentrations of total serum IgE were evaluated by ELISA as follows. Ninety-six-well Nunc Maxisorp plates (Nalge Nunc International, Rochester, NY, USA) were incubated for 1 to 1.5 hours with 50μL/well of PBS containing 7.5μg/mL goat anti-rat IgE (Immunology Consultant Laboratory, Portland, OR, USA). Plates were washed three times with 200μL/well of PBS, blocked with 200μL/well of PBS with 0.1%BSA (PBS-BSA) and incubated for 1 hr at room temperature. Plates were washed with 200μL/well PBS and incubated overnight in 200μL/well PBS at 4°C. Serum samples were thawed and diluted with PBS-BSA and added at 50μL/well. The serum from one rat was selected as a standard and was used on each plate. After incubating for 3 hrs at 4°C, the wells were washed and alkaline phosphatase-labeled goat anti-rat IgG (whole molecule) (Sigma-Aldrich, St. Louis, MO, USA), was added at 50μL/well. The plates were incubated for 1 hr at room temperature and washed 6 times with PBS. Plates were developed with 0.5mM MgCl_2_ and NaN_3_ in a 100 mM diethanolamine buffer at pH 9.5. The absorbance at 405 nm was measured.

Relative concentrations of peanut-specific IgE were determined in a similar manner, except that a) the wells were initially coated with peanut extract diluted in PBS (~10μg/mL), and b) goat anti-rat IgE and the alkaline phosphatase-labeled rabbit anti goat-IgG whole molecule (Sigma-Aldrich), were used instead of alkaline phosphatase-labeled goat anti-rat IgG.

Relative concentrations of FITC-specific IgG were determined in a similar manner, except that the wells were initially coated with FITC-BSA diluted in PBS (~10μg/mL), and that alkaline phosphatase-labeled goat anti-rat IgG (γ-chain specific) (Kirkegaard & Perry, Gaithersburg, MD, USA) was used.

Relative concentrations of DNP-specific IgM and IgG were determined in a similar manner, except that the wells were initially coated with DNP-human serum albumin (DNP-HSA) (Sigma-Aldrich) diluted in PBS (~10μg/mL), and that alkaline phosphatase-labeled goat anti-rat IgM (μ) (Kirkegaard & Perry) and alkaline phosphatase-labeled goat anti-rat IgG (γ-chain specific) were used, respectively.

Relative concentrations of HSA-specific IgM and IgG were determined in a similar manner except that the wells were initially coated with HSA (Sigma-Aldrich) rather than BSA.

Relative concentrations of DNP-specific IgG1, IgG2a, IgG2b, and IgG2c were determined in a similar manner except that a) the wells were initially coated with DNP-Albumin (Sigma- Aldrich) diluted in PBS, and b) the appropriate biotin labeled mouse anti-rat IgG subclass antibody (BD Biosciences) and alkaline phosphatase-labeled Streptavidin (BD Biosciences, Franklin Lakes, NJ, USA) at a 1:500 dilution, were used before adding the diethanolamine buffer developer.

### Preparation of rat muscle tissue extracts

To provide autologous antigens that would be recognized by natural antibodies in rat serum, a rat muscle tissue extract was prepared. For this purpose, rat muscle tissue was harvested from the thigh of a euthanized male WKY rat. Muscle tissue was flash frozen in liquid nitrogen and pulverized while frozen using a BioPulverizer. The resulting muscle tissue powder was thawed and then washed twice at 4°C. Each wash was performed by suspension of the tissue powder in 4.35 mL PBS/g tissue, mechanical agitation and mixing of the suspension, centrifugation of the suspension at 90 x g for approximately 1 minute, and disposal of the supernatant. After both washes were completed, additional PBS was added to bring the suspension to a total volume of 5.56 mL/g of muscle tissue. This suspension was homogenized using a homogenizer (OMNI GLH) with 2 pulses of approximately 45 seconds each, to lyse cell membranes and expose intracellular antigens. The homogenized suspension was spun once at 9,710 x g for 30 minutes at 4°C, and the supernatant, having a protein concentration of about 4.68 mg/mL, determined using a DC Protein Assay Kit (Bio-Rad Laboratories, Hercules, CA, USA) was collected and mixed with 10% glycerol. The muscle tissue extract was aliquotted, flash frozen in liquid nitrogen, and stored at -80°C.

### Binding of Immunoglobulin to muscle antigens as determined by immunoblotting

The antigen mixture for immunoblotting was prepared by mixing 280 μL muscle extract, 108 μL Novex NuPAGE LDS sample buffer (4x) (Life Technologies, Carlsbad, CA, USA), and 43 μL Novex NuPAGE sample reducing agent (10x) (Life Technologies) containing 500 mM dithiothreitol. The mixture was vortexed, boiled for 7 minutes at 100°C, vortexed again, and centrifuged for 7 minutes at 15,996 x g. Two hundred μL of the antigen mixture was loaded onto each 4 to 12% acrylamide gradient preparation gel (Novex NuPAGE 4–12% Bis-Tris ZOOM Gel, Life Technologies). Five μL of a molecular weight standard (PageRuler Plus, Thermo Scientific) was loaded into the standard well of each gel. Proteins were separated by electrophoresis at 70V for 3 hours. Antigens were transferred to PVDF membranes for 7 minutes at 20V using an iBlot gel transfer device (Ethrog Biotechnologies Ltd., Invitrogen) and iBlot mini gel transfer stacks (Life Technologies). PVDF membranes were blocked for 70 minutes at room temperature using 1.0% bovine serum albumin, 0.1% Tween 20, and 0.01% sodium azide in Tris buffered saline (blocking buffer). Each membrane was then cut into 16 strips (excluding the standard strip) approximately 4 mm wide. Fifteen strips were incubated overnight at 4°C with specific rat sera diluted 1/400 in blocking buffer, while 1 strip was incubated overnight in blocking buffer to act as a control for secondary antibody-conjugate binding (see below) to muscle antigens. Rat sera from biome depleted laboratory rats (n = 7) and biome enriched rats (n = 8) weighing greater than 250 grams were selected at random for the study.

After overnight incubation in rat sera, membrane strips were washed 3 x 10 minutes with Tris buffered saline. Strips were then incubated either for 1 hour at room temperature in alkaline-phosphatase conjugated, affinity purified goat antibody specific for the Fc region of rat IgG (Sigma-Aldrich), diluted 1/1000 in blocking buffer, to detect natural IgG antibody binding or for 1 hour at room temperature in alkaline-phosphatase conjugated, affinity purified goat antibody specific for rat μ-chain (Sigma-Alrich), diluted 1/1000 in blocking buffer, to detect natural IgM binding. Strips were then washed 3 x 10 minutes with Tris buffered saline and developed with 1-Step NBT-BCIP (nitro blue tetrazolium and 5-bromo-4-chloro-3-indolyl-phosphate) (Thermo Scientific). Strips that had been incubated in goat anti-rat IgG were developed for 28 minutes with fresh developer added after 14 minutes, and strips that had been incubated in goat anti-rat IgM were developed for 7 minutes. Finally, all strips were washed with distilled water twice for 6 minutes each, gently blotted with filter paper (to remove large water droplets), and air dried. Two membranes were utilized in this fashion for analysis of each antigen, one membrane for assessment of IgM binding, and another, run at the same time, for IgG binding.

Membranes were analyzed using Quantity One software v. 4.6.6 (Bio-Rad Laboratories) to quantify the amount of natural antibody binding to antigens on each strip. Lane background was subtracted using a rolling disk of radius 133 for strips incubated in goat anti-rat IgG, and a rolling disk radius of 100 for strips incubated in goat anti-rat IgM, to adjust for noise caused by natural antibody and anti-IgG or anti-IgM conjugate binding to the blocking buffer. All IgG bands were detected using a sensitivity of 50.5 and a noise filter of 4.4, and all IgM bands were detected using a sensitivity of 23.6 and a noise filter of 4.0. After all bands had been quantified, the amount of binding for each band on the control strip was subtracted from the corresponding bands on all the other strips, to account for anti-IgG and anti-IgM conjugate binding to muscle-derived antigens.

### Statistical analyses

Results were evaluated to determine if the data were normally distributed using the D’Agostino and Pearson omnibus normality test. Isotype concentrations from biome depleted and biome enriched animals were compared using a t-test if the data passed the normality test, and a Mann-Whitney test if the data did not pass the normality test. Multiple subclasses of IgG were evaluated using a 2-way ANOVA, followed by post-hoc t-tests or Mann-Whitney tests, where appropriate. GraphPad Prism Version 5.01 (GraphPad Software, Inc., San Diego, CA) was utilized for all statistical calculations.

## Results

### Body weights of biome depleted and biome enriched rats

**Fig 1:**
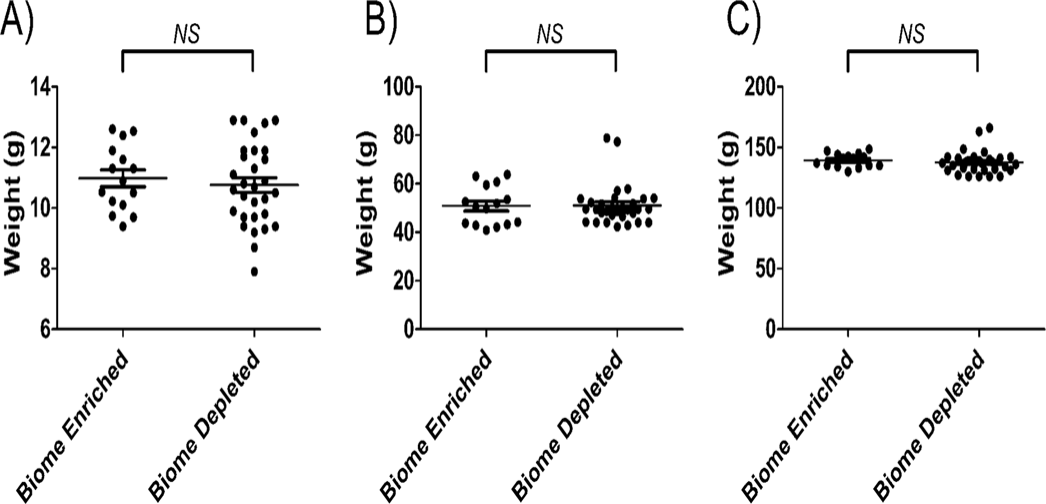
Body weights of biome depleted and biome enriched rats at (A) 4 days old, (B) 23 days old, and (C) 63 days old. The mean ± the standard error is indicated by the horizontal lines. No statistical significance (NS) was observed with comparing data from biome depleted and biome enriched animals using a t-test.

All F_1_ rats exhibited normal respiration and social interactions. In addition, body weights of all F_1_ rats were measured when the rats were 4 days old, 23 days old and again at 63 days old to gauge the effect of biome depletion and enrichment on the overall health of the animals. As shown in Fig. 1, no significant difference was found between the mean body weights of the biome depleted and the biome enriched animals. However, at 23 days of age, two of the biome depleted animals showed a 40% greater body mass than average for either group. Two biome depleted animals of unusually high weight were also present at day 63, although it is unknown if these were the same two animals, and their weights were not as high above the mean at day 63 as on day 23.

### Total serum IgE and anti-peanut IgE levels in biome depleted and biome enriched rats

**Fig 2:**
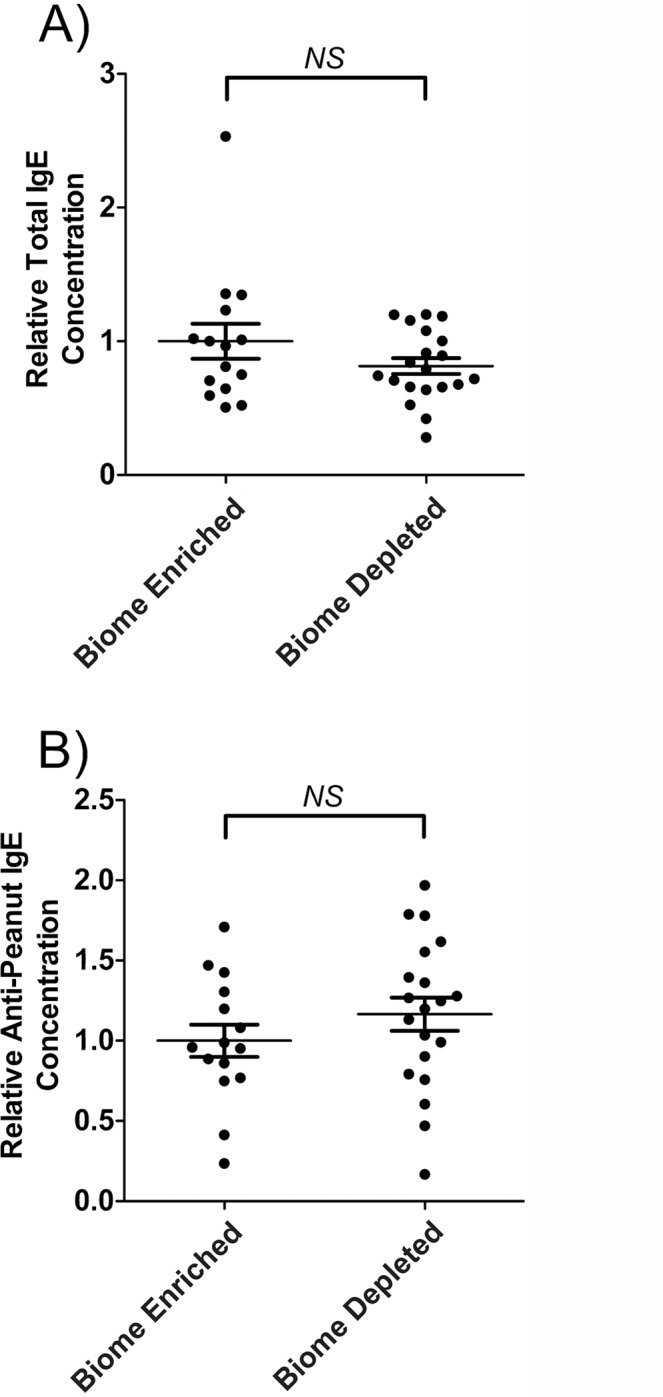
Relative concentration of IgE and of peanut-specific IgE in the serum of biome depleted (n = 20) and biome enriched (n = 15) rats following immunization. The relative concentration of antibody was determined by ELISA as described in the Methods. The means and standard errors are indicated by the horizontal bars, and the data were assessed using a t-test. (A) Following immunization, no statistically significant difference (NS) was observed when comparing total serum IgE levels from biome depleted and biome enriched animals. In addition, (B), following immunization, no statistically significant difference (NS) was observed when comparing anti-peanut IgE levels from biome depleted and biome enriched animals.

As shown in Fig. 2A and in Table 1, no statistically significant difference was observed between serum IgE levels in biome depleted versus biome enriched rats following immunization. Similarly, no statistically significant difference was observed between anti-peanut IgE levels in biome depleted versus biome enriched rats following immunization (Fig. 2B).

**Table 1:** Effect of biome depletion on the humoral immune response.

Antibody	Biome Enriched	Biome Depleted	*p*-value^1^
Total IgE	1.00 ± 0.51	0.81 ± 0.26	^b^0.38
Peanut-specific IgE	1.00 ± 0.39	1.17 ± 0.47	^a^0.27
FITC-specific IgG	1.00 ± 0.40	0.59 ± 0.35	^b^ **0.014**
DNP-specific IgM	1.00 ± 0.11	0.76 ± 0.12	^b^ **<0.0001**
DNP-specific Total IgG	1.00 ± 0.45	0.70 ± 0.29	^b^ **0.039**
DNP-specific IgG1	1.00 ± 0.34	1.12 ± 0.43	^b^0.34
DNP-specific IgG2a	0.60 ± 0.34	0.46 ± 0.47	^b^ **0.037**
DNP-specific IgG2b	0.48 ± 0.22	0.24 ± 0.22	^b^ **.0012**
DNP-specific IgG2c	2.00 ± 0.43	1.83 ± 0.43	^b^0.43
HSA-specific IgM	1.00 ± 0.21	1.11 ± 0.21	^**a**^0.13
HSA-specific IgG	1.00 ± 0.78	0.38 ± 0.28	^b^ **0.0015**
Muscle-specific IgM	1.00 ± 0.07	0.67 ± 0.08	^a^ **0.0054**
Muscle-specific IgG	1.00 ± 0.09	0.72 ± 0.06	^a^ **0.028**

Antibody levels were determined by ELISA, except anti-muscle antibodies, which were determined by immunoblotting. The means and standard deviations are shown.

^1^ The p-values were determined using a t-test for normally distributed data indicated by ^a^ or a Mann-Whitney test for non-normally distributed data indicated by ^b^ (see Methods). Significant values (< 0.0500) are shown in **bold**. The *p*-values shown for IgG subclasses are post hoc tests run after a 2-way ANOVA that showed no significant effect of the state of the biome on IgG subclass concentrations.

### Anti-FITC IgG in the serum of biome depleted and biome enriched rats

**Fig 3:**
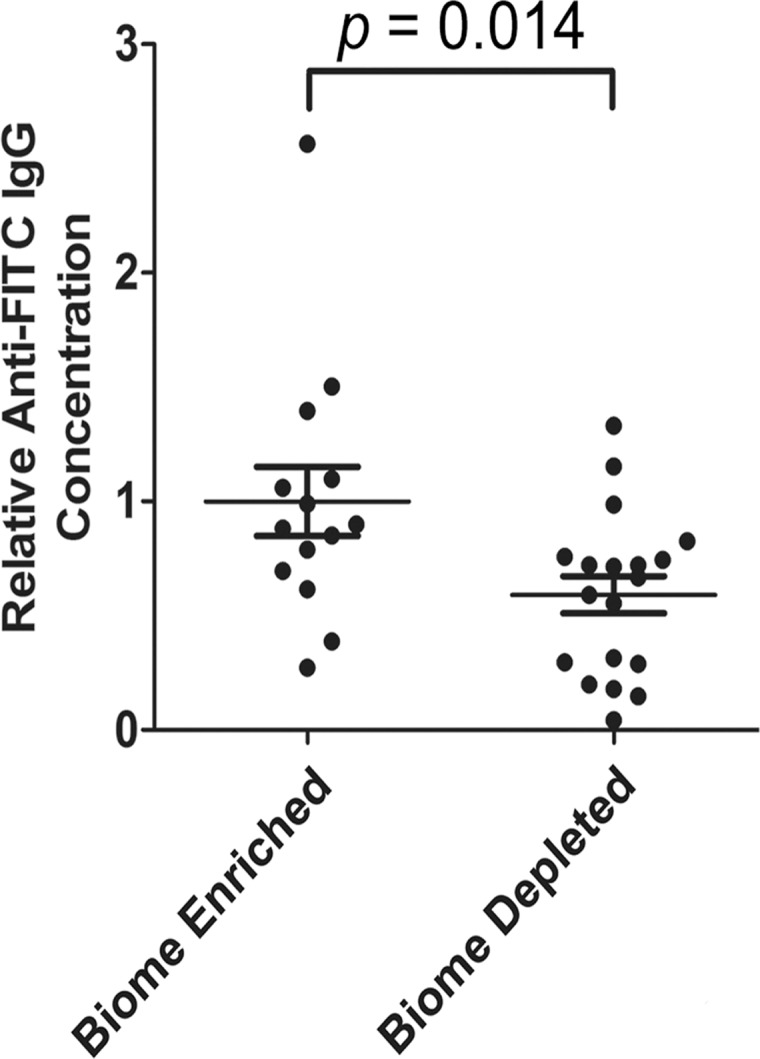
Relative concentration of FITC-specific IgG in the serum of biome depleted (n = 20) and biome enriched (n = 15) rats. The relative concentration of antibody was determined by ELISA as described in the Methods. The means and standard errors are shown. No statistical significance (NS) was observed with comparing data from biome depleted and biome enriched animals using a t-test.

The level of anti-FITC IgG in serum following immunization of biome depleted and biome enriched animals was assessed as a measure of the immune response against FITC-KLH, a T-dependent antigen. The mean concentration of anti-FITC IgG in biome depleted animals was reduced by 41% compared to that in biome enriched animals following immunization (p = 0.014) (Fig. 3).

### Anti-DNP IgM and IgG in the serum of biome depleted and biome enriched rats

**Fig 4:**
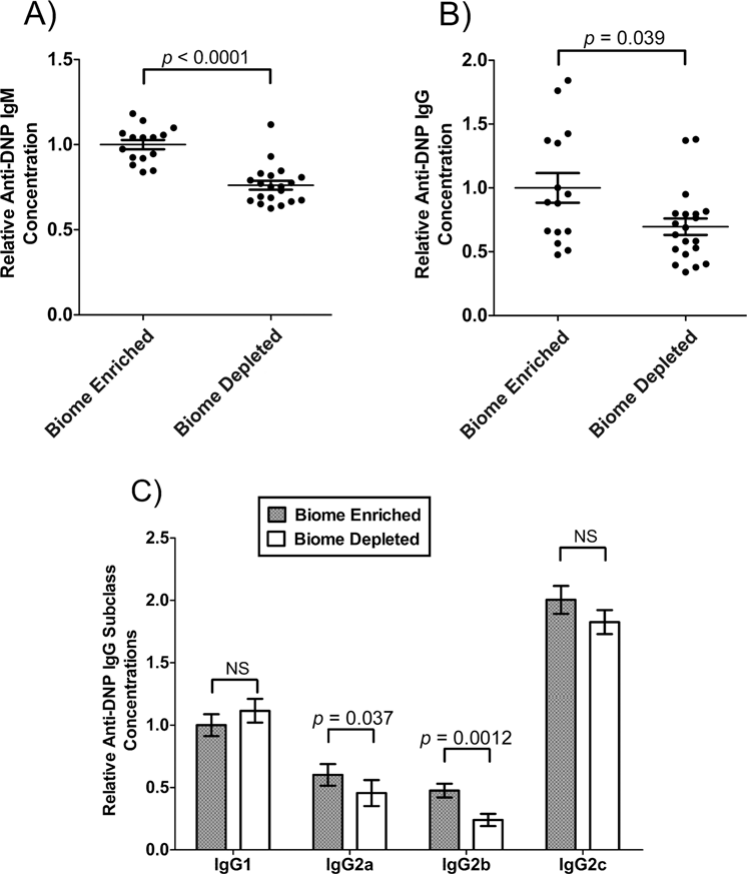
Relative concentration of DNP-specific antibody in the serum of biome depleted (n = 20) and biome enriched (n = 15) rats. The relative concentration of antibody was determined by ELISA as described in the Methods. Relative levels of (A) IgM, (B) IgG, and (C) subclasses of IgG are shown. The means and standard errors are shown. The *p*-values associated with comparing data from biome depleted and biome enriched animals using a t-test are shown. (NS = not significant)

The level of anti-DNP IgM and IgG in serum following immunization of biome depleted and biome enriched animals was assessed as a measure of the immune response against DNP-Ficoll, a T-independent antigen. The mean concentrations of anti-DNP IgM (Fig. 4A) and anti-DNP IgG (Fig. 4B) were significantly greater in biome enriched animals compared to biome depleted animals (p < 0.0001 and p = 0.039, respectively) following immunization.

The relative levels of anti-DNP IgG1, IgG2a, IgG2b, and IgG2c in the serum of biome depleted (n = 20) and biome enriched (n = 15) rats following immunization are shown in Fig. 4c. Although the 2-way ANOVA showed no significant effect of the state of the biome on the results, post-hoc tests showed significant effects on IgG2a concentrations (p = 0.037) and IgG2b concentrations (p = 0.0012), with biome enriched rats having mean levels approximately 30% greater and 2-fold greater, respectively, than biome depleted rats. Further, mean levels of anti-DNP IgG1 were actually decreased in biome enriched rats. Although this decrease was not statistically significant, it does strongly suggest that biome depletion and enrichment does not affect all aspects of the humoral response equally.

### Levels of “natural” IgM and IgG antibodies in biome depleted and biome enriched animals

**Fig 5:**
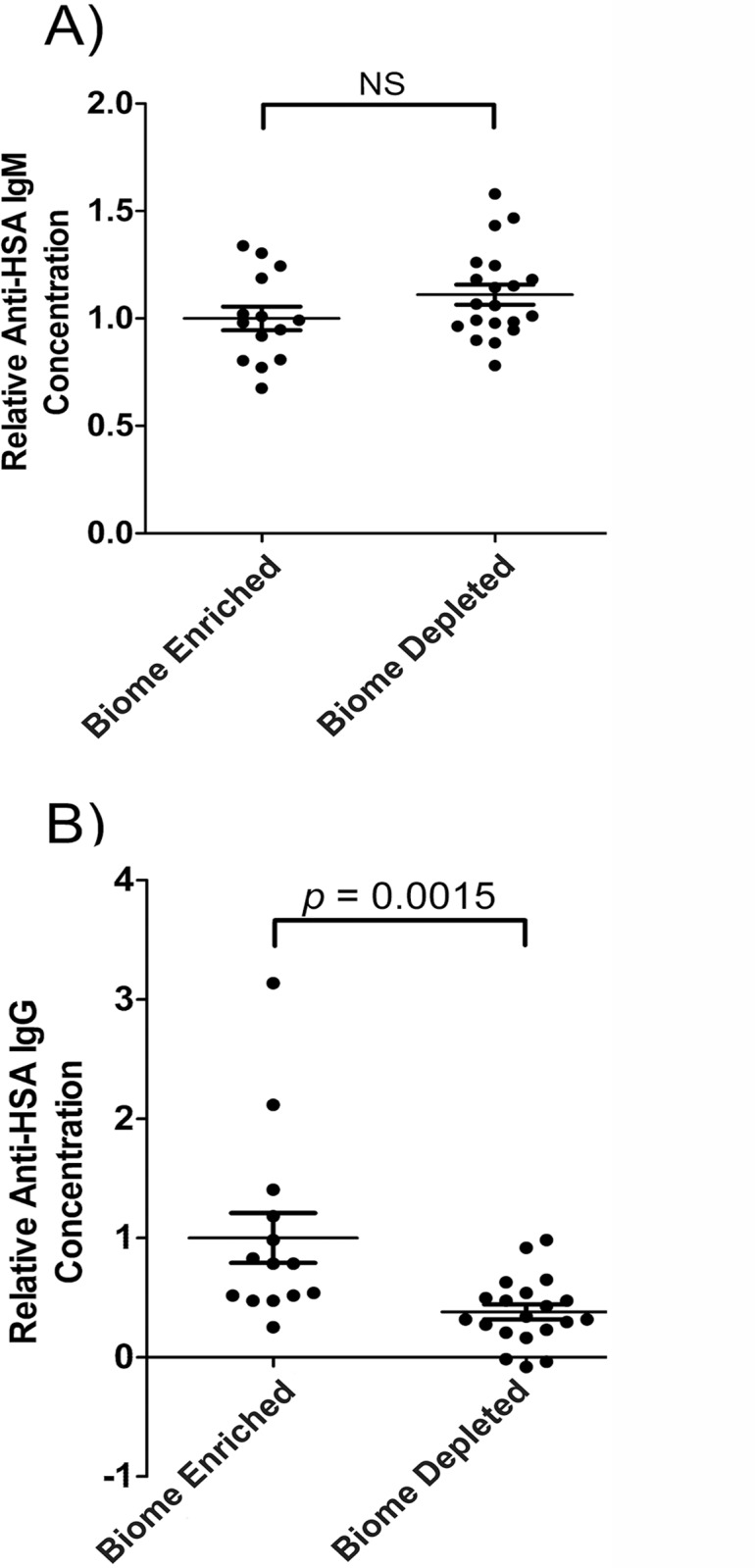
Natural anti-human serum albumin antibody levels in the serum of biome depleted (n = 20) and biome enriched (n = 15) rats. The relative concentration of antibody was determined by ELISA as described in the Methods. Relative levels of (A) IgM and (B) IgG are shown. Binding to human serum albumin (HSA) was used as a measure of reactivity toward a xenogeneic antigen for which the animals lacked previous exposure. The means, standard errors, and the *p*-values associated with comparing data from biome depleted and biome enriched animals using a t-test are shown. (NS = not significant)

The levels of natural antibodies, antibodies that are present without a known history of sensitization, were evaluated in biome depleted and biome enriched animals. For this purpose, two antigens were selected, one xenogeneic (human serum albumin; HSA) and one autologous (rat muscle tissue extract). Typically, antibodies in normal (non-diseased) rats which bind to these antigens are polyreactive (broadly reactive) antibodies that are the first line of defense against foreign antigens or damaged tissue, and do not react strongly with intact tissues[10–15]. Levels of both IgM and IgG natural antibodies were evaluated. Differences observed in natural antibody levels as a function of biome enrichment were somewhat dependent on isotype, with levels of anti-HSA IgG but not anti-HSA IgM being significantly affected by biome enrichment (Fig. 5). The decrease in anti-HSA antibodies associated with biome depletion was particularly notable, with those animals having less than 40% of the level of antibody found in biome enriched animals (p = 0.0015; Fig. 5).

**Fig 6:**
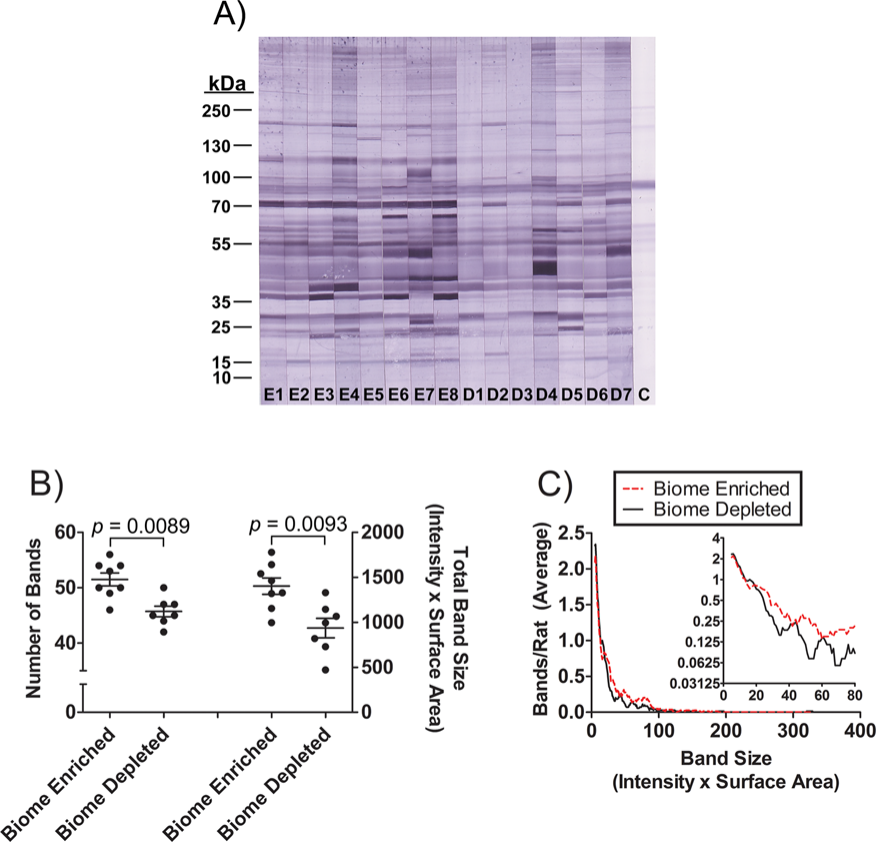
Binding of natural anti-rat muscle IgM in the serum of biome depleted and biome enriched rats as evaluated by immunoblotting. (A) Rat muscle extracts were separated by SDS PAGE and probed by immunoblotting as described in the Methods. The analysis was limited to 15 animals (n = 8 biome enriched; lanes E1 through E8, and n = 7 biome depleted; lanes D1 through D7) due to size constraints of the gel. A control strip with no serum is labeled “C”, and indicates reactivity of the anti-IgM conjugate with muscle-derived antigens. (B) The number of bands recognized by natural IgM in individual sera (p = 0.0089) and the total reactivity of natural IgM from each serum sample (p = 0.0093) are shown, with the bars indicating the mean and standard error. (C) The distribution of bands as a function of band size is shown. For this analysis, the average number of bands in biome depleted and biome enriched rats (Y-axis) was plotted on linear and log scales (main figure and figure inset, respectively) versus different band sizes (X-axis).

**Fig 7:**
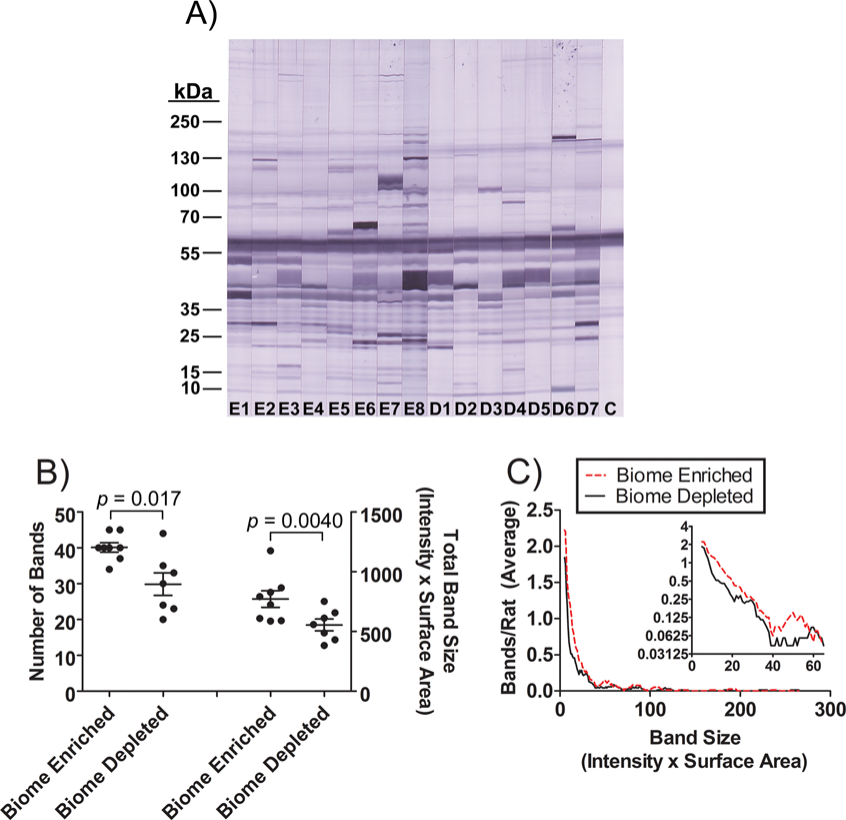
Binding of natural anti-rat muscle IgG in the serum of biome depleted and biome enriched rats as evaluated by immunoblotting. (A) Rat muscle extracts were separated by SDS PAGE and probed by immunoblotting as described in the Methods. The analysis was limited to 15 animals (n = 8 biome enriched; lanes E1 through E8, and n = 7 biome depleted; lanes D1 through D7) due to size constraints of the gel. A control strip with no serum is labeled “C”, and indicates reactivity of the anti-IgG conjugate with muscle-derived antigens. (B) The number of bands recognized by natural IgG in individual sera (p = 0.017) and the total reactivity of natural IgG from each serum sample (p = 0.040) are shown, with the bars indicating the mean and standard error. (C) The distribution of bands as a function of band size is shown. For this analysis, the average number of bands in biome depleted and biome enriched rats (Y-axis) was plotted on linear and log scales (main figure and figure inset, respectively) versus different band sizes (X-axis).

The repertoire of natural antibodies recognizing autologous antigens (rat muscle tissue extract) in biome enriched and biome depleted animals was evaluated by Western blotting (Figs. 6 and 7). The results suggest that the concentration of natural antibodies is dramatically increased by biome enrichment. The binding of IgM to autologous antigens (Fig. 6) was characterized by a moderate (11%) difference in the average number of antigens recognized (Fig. 6B), but given the high density of bands, it is possible that the presence of overlapping bands may have caused an underestimation of the bands recognized, particularly in the lanes with high intensity of binding. Biome depleted animals had about 34% less binding of IgM to autologous antigens, on average, and a distribution analysis showed enhanced binding of IgM from biome enriched animals to both high intensity and low intensity bands on the membranes (Fig. 6C). These results suggest that the increase in IgM binding to autologous antigens as a result of biome enrichment may be due to both increased concentrations of antibody and an increased range of specificity of the repertoire. Similar results were obtained when examining the IgG repertoire (Fig. 7), again suggesting that the increase in IgG binding to autologous antigens as a result of biome enrichment may be due to both increased concentrations of antibody and increased range of specificity of the repertoire.

## Discussion

The biome enrichment protocol employed was directed primarily at the biome of parental rats, with the F1 rats receiving less stimulation, as described in the Methods. For example, the F0 rats but not the F1 rats received regular exposure to bedding from the cages of wild-caught rats. This protocol simulated, in part, the fact that newborn F1 rats are not, in the wild, extensively exposed to new components of the biome except through contact with the mother. However, F1 rats were housed in the same room as the wild rodents and, until weaning, in the same cages as the F0 rats, so extensive enrichment of the biome in the F1 rats may have occurred. That being said, the immune response of the F1 rats could have been extensively altered by transmission of immune components through the mother’s milk and by epigenetic effects. With this in mind, future studies might initiate biome enrichment after birth to probe the extent to which mother-to-offspring transmission of immune components is important.

The results demonstrate that biome enrichment enhances immune responsiveness under the conditions used in this study, which employed vaccination with adjuvant. Although this study is clearly an experiment involving enrichment of the biodiversity of laboratory animals, the study has implications for humans in a Westernized, biome depleted environment. These studies suggest that biome depletion may be associated with attenuated humoral immune responses to both T-independent and T-dependent antigens. To the extent that biome depletion affects laboratory animals and humans in Western countries in a similar fashion, the implications of this finding are potentially far-reaching. For example, attenuated responses to tumor antigens as a result of biome depletion might underlie, at least in part, the proposed connection between increased rates of cancer and biome depletion[16]. Further, decreased levels of “natural” IgG and IgM observed in biome depleted (laboratory) environments could exacerbate the problem, since the natural antibody repertoire is involved in tumor surveillance [6, 7]. In this manner, decreased tumor surveillance in biome depleted environments could promote cancer progression and operate synergistically with biome depletion-associated inflammation, a potential initiator and promoter of carcinogenesis [16, 17].

Studies comparing wild and laboratory rats [5] have suggested the importance of biodiversity in the development of the natural antibody repertoire. These studies extend that work considerably, emphasizing the potential importance of biome enrichment for effective function of the humoral immune system and giving traction to the idea of biome enrichment for all Westernized humans. Such enrichment in the clinical setting might conceivably utilize a wide array of eukaryotic organisms, including protozoans and various symbiotic flatworms and roundworms. However, for practical reasons that involve the desire for highly controlled reintroduction of organisms into the population with little or no adverse side effects [18, 19], most studies in animal models and especially in humans have focused on the reintroduction of helminths, worms that live in the intestine, into the body’s ecosystem. The findings in this study suggest that biome enrichment with such organisms as helminths, which reduces a wide range of allergic and autoimmune conditions[20], is not necessarily “immunosuppressive”. Rather, this study suggests that biome enrichment, perhaps including helminth therapy, might be an “immune trainer”, and thus fundamentally different in principle and practice than drugs aimed at averting disease via suppression of immune function.
